# Supplementary material for: Integration of background knowledge for automatic detection of inconsistencies in gene ontology annotation
Source: Bioinformatics. 2024 Jun 28;40(Suppl 1):i390–400. doi: 10.1093/bioinformatics/btae246 (PMC11256942; doi:10.1093/bioinformatics/btae246)
Supplement: btae246_Supplementary_Data [file btae246_supplementary_data.pdf]

Bioinformatics
doi.10.1093/bioinformatics/xxxxxx
Advance Access Publication Date: Day Month Year
Manuscript Category
OXFORD

Data and text mining

Integration of Background Knowledge for Automatic Detection of Inconsistencies in Gene Ontology Annotation

Jiyu Chen [1,3]*, Benjamin Goudey [1], Nicholas Geard [1] and Karin Verspoor [2,]*

[1] School of Computing and Information Systems, The University of Melbourne, Parkville, 3010, Australia
[2] School of Computing Technologies, RMIT University, Melbourne, 3000, Australia.
[3] Data61, The Commonwealth Scientific and Industrial Research Organisation, Marsfield, 2122, Australia.

*To whom correspondence should be addressed.
Associate Editor: Not Applicable

Abstract

Motivation: Biological background knowledge plays an important role in the manual quality assurance (QA) of biological database records. One such QA task is the detection of inconsistencies in literature-based Gene Ontology Annotation (GOA). This manual verification ensures the accuracy of the GO annotations based on a comprehensive review of the literature used as evidence, Gene Ontology (GO) terms, and annotated genes in GOA records. While automatic approaches for the detection of semantic inconsistencies in GOA have been developed, they operate within predetermined contexts, lacking the ability to leverage broader evidence, especially relevant domain-specific background knowledge. This paper investigates various types of background knowledge that could improve the detection of prevalent inconsistencies in GOA. Additionally, the paper proposes several approaches to integrate background knowledge into the automatic GOA inconsistency detection process.
Results: We have extended a previously developed GOA inconsistency dataset with several kinds of GOA-related background knowledge, including GeneRIF statements, biological concepts mentioned within evidence texts, GO hierarchy and existing GO annotations of the specific gene. We have proposed several effective approaches to integrate background knowledge as part of the automatic GOA inconsistency detection process. The proposed approaches can improve automatic detection of self-consistency and several of the most prevalent types of inconsistencies.
Conclusion: This is the first study to explore the advantages of utilizing background knowledge and to propose a practical approach to incorporate knowledge in automatic GOA inconsistency detection. We establish a new benchmark for performance on this task. Our methods may be applicable to various tasks that involve incorporating biological background knowledge.
Availability: `https://github.com/jiyuc/de-inconsistency`.

1 Introduction

Literature-based gene ontology annotations (GOA) (Carbon *et al.*, 2021) are biological database records that provide associations between genes and information about their molecular functions, biological processes, and cellular locations, described utilizing Gene Ontology (GO) terms, and derived from evidence found in the scientific literature (Ashburner *et al.*, 2000). The GO provides a standardized and hierarchical description of gene function. GOA can be used for a range of biological research tasks, such as gene-category analysis (Bauer, 2017), gene enrichment analysis (Huang *et al.*, 2009), and drug discovery (Haendel *et al.*, 2018).

Several quality issues have been identified in GOA, including inconsistency, duplication, and errors (Carbon *et al.*, 2021; Bateman *et al.*, 2021; Bult *et al.*, 2019; Chen *et al.*, 2017b,a). Faria *et al.* (2012) found orthologous and homologous genes contain inconsistent GOA in the UniProtKB database (Bateman *et al.*, 2021). A large proportion of inconsistent GOA are associated with broad GO terms located at higher levels of the Gene Ontology direct acyclic graph (DAG) (Camon *et al.*,

© The Author 2015. Published by Oxford University Press. All rights reserved. For permissions, please e-mail: journals.permissions@oup.com

2005; Škunca *et al.*, 2012; Poux and Gaudet, 2017), which results in insufficient specificity in describing gene function information. Chen *et al.* (2021) found that certain instances of inconsistent GOA were linked to irrelevant GO terms. These terms were identified as keywords within the direct literature evidence, but did not describe the specific gene's molecular activity, cellular compartment, or biological function. Through an analysis of different guidelines from biological databases and oral communication with expert curators, Chen *et al.* (2022) established four main types of inconsistencies related to GOA.

The GO curation community has prioritized the review of GOA consistency in their routine quality maintenance procedures (Carbon *et al.*, 2021). Compared to automatic approaches, manual detection of inconsistency in GOA is considered more reliable due to humans' comprehensive understanding of biological background knowledge. However, manual approaches for addressing inconsistencies in GOA can be both time-consuming and resource-intensive, relying on the expertise of curators in both biology and the scientific literature, as well as comprehensive guidelines (Balakrishnan *et al.*, 2013; Poux and Gaudet, 2017; Thomas, 2017). Furthermore, these manual approaches cannot scale up with the rapidly evolving knowledge related to gene function. As a potential solution, automatic approaches incorporating human-in-the-loop curation have been proposed (Chen *et al.*, 2021, 2022).

One existing automatic approach to GOA inconsistency detection (Chen *et al.*, 2022) is not yet practical as it fails to consider the complexity and variability of biological information, such as the linguistic variation in the expression of biological concepts in evidence text (e.g., *apoptosis* vs. *cell death*), particularly where this variation extends beyond simple synonymy. This deficiency relates to its limited use of background knowledge, relying only on the title and abstract of direct literature, which does not fully capture the complexity of or contexts for GOA.

GOA are important resources for supporting modern biological research. However, GOA are subject to inconsistencies such as the selection of overly broad or specific GO terms or incorrect association to gene mentions. These inconsistencies are problematic because they can influence the reliability of downstream biological research tasks or cause cascading errors in biological databases (Gilks *et al.*, 2002; Goudey *et al.*, 2022). Existing automatic approaches for detecting inconsistencies within GOA are constrained in their real-world applicability due to their limited use of relevant background knowledge. To address this issue, we investigate several types of background knowledge related to GOA and propose integration methods to improve the automatic detection of GOA inconsistencies.

Biological background knowledge plays an important role in GOA curation. Taking this instance into consideration. The evidence text reads as follows: "RHGF-2 RhoGEF activity is specific to the C. elegans RhoA homolog RHO-1 as determined by direct binding, GDP/GTP exchange, and serum response element-driven reporter activity" (PMID:22363657). In a supported annotation, the GO term "GDS (GO:0005085)" is erroneously assigned to the wrong gene"RHO-1", the misidentified gene is mentioned alongside the correct gene "RHFG-2" in the presented evidence text. Human curators can easily spot the misidentified gene with reference to the GO DAG and existing annotation. Here, "GTP binding" (GO:0005525) is an existing annotation to the gene "rho-1" which is positively regulated by "GDS" (GO:0005085), referring to GO DAG. Thus, the function "GDS" is only possible to occur in the related gene"RHGF-2", co-mentioned in the evidence. However, such kind of latent constraints cannot be solely captured from reading direct evidence of a single GOA instance without referring to the GO DAG. In fact, many GO annotation patterns have been discovered and utilized as background knowledge to support the quality assurance. For example, the reference of GO constraints to taxons (Huntley *et al.*, 2014) which have helped the correction of 1.6 million inconsistent annotations (Dimmer *et al.*, 2010). In short, a thorough understanding of background knowledge related to GOA is critical for facilitating practical automatic GOA inconsistency detection.

To the best of our knowledge, this is the first empirical investigation of approaches for integrating background knowledge to facilitate automated inconsistency detection in real-world GOA datasets. We make three key contributions:

- We find integrating background knowledge related to the GOA can lead to a substantial improvement in the performance of GOA inconsistency detection. As a result, our developed approaches have achieved a new state-of-the-art in this area.
- We establish a framework to investigate various approaches for integrating different types of background knowledge in order to facilitate automated detection of GOA inconsistencies.
- We extend an existing dataset (Chen *et al.*, 2022) by sourcing and adding GOA-related background knowledge to each instance, such as using GeneRIF statements as alternative evidence text(Brown *et al.*, 2015), encoding existing GO annotations patterns obtained from QuickGO (Binns *et al.*, 2009), encoding GO hierarchical knowledge using GO DAG, and other relevant sources. We substitute the test set in the original dataset (Chen *et al.*, 2022) using GOA instances directly sampled from real-world databases.

2 Background

The GOA inconsistency detection task was first proposed by Chen *et al.* (2021, 2022), aiming to detect GOA self-consistency and four primary types of inconsistencies at record level, including over-broad, over-specific, irrelevant GO mention, and incorrect selection of gene mention. These (in)consistencies are modeled as typed semantic relationships in a triplet of GO term, gene product, and naturally written evidence. We follow the same multi-class single-output classification setting to detect typed inconsistencies. In this framework, the classifier determines whether a given GOA triplet is self-consistent or not and identifies the type of inconsistency reflected in the instance. Our approach involves integrating GOA-related background knowledge into the same modeling framework.

Below, we summarise several types of GOA-related background knowledge, which are commonly used by human curators to assess GOA quality. Identifying effective methods to integrating this background knowledge could significantly improve methods to automatically detect GOA inconsistencies.

Gene Reference Into Function (GeneRIF) statements provide a concise summary of key gene function knowledge written in natural language (Brown *et al.*, 2015). Although their original purpose was to improve the efficiency of manual evidence review, they have been repurposed to facilitate the automated extraction of gene-disease associations, surpassing Online Mendelian Inheritance in Man (OMIM) database (Hamosh *et al.*, 2005) as the source for tracing gene-disease relationships. There are strong semantic connections between GO and GeneRIF (Lu *et al.*, 2006). The findings of a study suggested that the identification of GO terms in the texts can aid automatic selection of candidate sentences for generating GeneRIF (Gobeill *et al.*, 2008). Therefore, it is worth investigating whether incorporating GeneRIF statements can improve the detection of inconsistencies in GOA.

Biological concepts are mentioned as words within evidence text. The identification of these concepts have helped the implementation of many automatic GOA production systems in the BioCreative IV task Mao *et al.* (2014). Since GOA capture the relationship between a gene product and a GO term, both of which are fundamental biological concepts, it is assumed that the identification of these concepts can facilitate the automated detection of inconsistencies in GOA. Currently, several systems

can be directly leveraged to extract biological concepts from PubMed articles. For instance, PubTator is capable of automatically identifying mentions of biological concepts from raw text (Wei *et al.*, 2019), whereas ConceptMapper (Tanenblatt *et al.*, 2010; Funk *et al.*, 2014), MetaMap (Aronson, 2001), and NCBO annotator (Jonquet *et al.*, 2009) can extract GO term mentions from unstructured text.

The hierarchical structure of the GO can be modelled as a directed acylic graph (DAG). Curators use the GO DAG to find GO terms with the most appropriate specificity for representing the function of a specific gene. Previous work proposed a graph neural network model to encode the specificity relationships on the GO DAG and improved the detection of GOA inconsistency caused by over-broad and over-specific selection of GO terms (Chen *et al.*, 2022). This study explores whether incorporating the GO DAG or other related knowledge, including the biological concept mentions, the GeneRIF statements, or existing GOA patterns, can improve the identification of inconsistencies in GOA.

Consistency evaluation of a single GOA instance often considers the full collection of existing GOA records that are associated with a curated gene. An indicator of potential inconsistency in GOA arises when a gene product is assigned multiple distinct GO terms that essentially describe similar gene functions. This suggests a potential issue if annotations for the same gene product are not uniform when characterizing similar gene functions. Such co-annotation patterns for a given gene (Carbon *et al.*, 2021) can help detect inconsistencies in GOA. In addition, having multiple annotators work on the same set of gene products is a strategy sometimes used to support error detection (Balakrishnan *et al.*, 2013).If there are discrepancies in the annotations made by different annotators, it indicates that there may be inconsistencies in the GOA database. Wood *et al.* (2020) utilized co-annotation patterns to develop heuristic rules aimed at detecting erroneous annotations across five species. Building on the idea of considering a collection of related annotations together, our work proposes automatic approaches for encoding the knowledge that is inherent in the existing GOA, with the aim of investigating its potential utility for automatic detection of inconsistencies in GOA.

3 Methods

We modelled GOA self-consistency or inconsistencies at the record level as typed semantic relationships in a triplet of GO term, gene product, and naturally written literature title and abstract as evidence. This approach extends prior work (Chen *et al.*, 2022) by integrating various kinds of background knowledge into each record. Our goal is to develop classification models that, for a given GOA triplet and related background knowledge as input, make a classification decision as to whether the triplet is self-consistent, and if not, what type of inconsistency is reflected in the instance. Background knowledge is integrated into the classification process in several different ways, such as replacing or augmenting the input evidence text, or by encoding the background knowledge and embedding within the classification framework. That is, the classifier makes a determination about whether the textual evidence supports the GO term annotation to the given gene product. To simplify our study, we assume that a GOA can only have one type of inconsistency and that the typed inconsistencies are independent of each other. The outline of our methods is illustrated in figure 1.

3.1 Data

We prepared the data by sourcing relevant background knowledge for each instances in the GOA inconsistency dataset published by Chen *et al.* (2022). The dataset contains three subsets with non-overlapping GOA instances that are independent to each other, including a training set, a development set, and a held-out test set. Each subset contains a balanced number of instances across the categories of self-consistency and four distinct type of inconsistencies, including over-specific (OS) or over-broad (OB) selection of GO terms, selection of irrelevant GO mentions (IM), annotation of incorrect gene product (IG). In total, there are 10,000 instances in the training set, 500 instances in the development set, and 2,500 instances in the test set. The original test set contained instances that were obtained from the BC4GO corpus (Van Auken *et al.*, 2014). However, after discussions with the creator of BC4GO from NCBI (C. H. Wei, personal communication, Jan 23, 2023), we found that these instances were not sufficiently reliable for our needs. Firstly, the evidence provided by BC4GO is annotated at the sentence level, which does not align with real-world scenarios where evidence is typically provided at the level of abstracts and full-text articles. Secondly, the annotation agreement in BC4GO for evidence text spans has only reached an F_1 of 42.7%. Referring to the annotated sentences as evidence may lead to a lack of sufficient supportive information for automatic GOA inconsistency detection models. Additionally, the corpus is limited in size, containing only 200 articles, and the annotations were conducted in the year 2014. As a result, we noticed some GOA instances have become outdated. Relying on these annotated sentences as evidence may not be sufficient to support the development of automatic GOA inconsistency detection models. Therefore, we replaced the original test set with a new version where instances were directly sampled from the NCBI database. Further details of this substitution are discussed in Section 5.

The dataset (Chen *et al.*, 2022) and the modified test set have been specifically designed to facilitate the investigation of automatic approaches with regard to their capability to discern the nuanced semantics of GO specificity. The over-specific instances in the dataset were constructed by utilizing directly connected children GO terms, while over-broad instances have been created using ancestor GO terms. As an ancestor may not be directly linked to a descendant on the GO DAG, it can have a greater semantic distance. Consequently, over-specific instances are generally more difficult for automatic semantic inference than over-broad instances.

3.2 Sourcing Background Knowledge related to GOA

GeneRIF Statements as alternative evidence text

We retrieved GeneRIF statements from NCBI as alternative context of evidence to GOA, replacing the literature title and abstract for each instance as evidence in the original dataset. GeneRIF statements typically comprise a single, manually created, short sentence that describes the essential gene function knowledge. Each GeneRIF statement is linked to its supporting literature, indexed by PMID and GeneID combined (PMG). All instances in the selected GOA dataset can be linked to a specific GeneRIF statement using PMG. In contrast, a GeneRIF statement may be linked to multiple GOA instances with shared PMG but different GO terms. For example, evidence from the literature (PMID:22343943) indicates that "(Gene:2475)" enables the cellular function of "protein serine/threonine kinase activity (GO:0004674)" and is involved in the biological process of "cellular response to starvation (GO:0009267)", creating two GOA instances. However, it should be noted that a GeneRIF statement is not necessarily guaranteed to contain the gene function information represented by the linked GOA. GeneRIF only describes a single piece of key gene function information in the direct literature, while the literature may contain multiple pieces of gene function information and support multiple GOA instances. Therefore, it is important to investigate the effectiveness of using GeneRIF as an alternative source of evidence.

Pre-extraction of biological concepts

To identify biological concepts. we applied PubTator (Wei *et al.*, 2019) to identify the token span of several kinds of biological concepts in the literature title and abstract, including disease, chemical compound, species, mutation, and cell line, and apply ConceptMapper (Funk *et al.*, 2014) to identify the token span of GO mention.

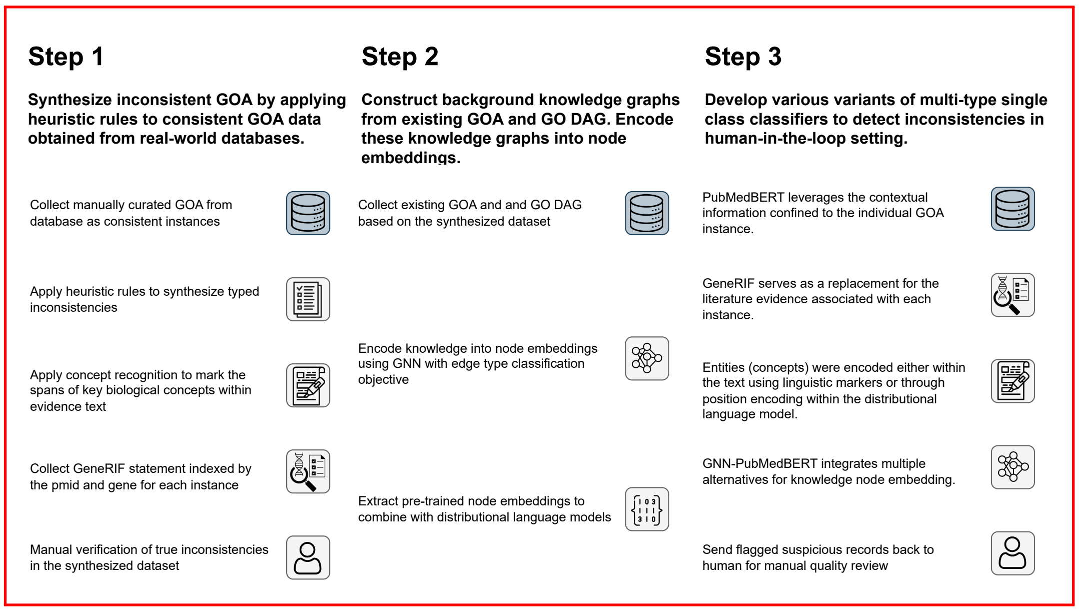

Fig. 1. Overview of the methodological framework, illustrating the stages of data collection and synthesis, background knowledge integration, and modeling. The human-in-the-loop evaluation in Step 3, crucial for formulating the GOA inconsistency detection framework, remains unexplored in the studies presented in this paper.

Pre-processing of GO Direct Acyclic Graph

The inference of the specificity of a single pair of GO terms cannot be achieved by leveraging the entire GO DAG, as it is unrealistic given the size of the graph. Consequently, we opted to select only a portion of the GO DAG that has direct connections to the GO terms present in the dataset. Utilizing QuickGO (Binns *et al.*, 2009), we retrieved the children of GO terms that are related either through the “is_a” or “part_of” relation types. GO terms in other relation types, such as “regulate” are not retrieved as they do not provide any GO specificity knowledge. We then reconstruct a sub-part of the GO DAG with vertices representing GO terms and edges representing directed hierarchical relations. This sub-graph is used for encoding GO specificity knowledge with a Graph Neural Network (GNN) introduced in Section 3.3. Direction-reversed edges with the “reverse” edge type are added to the graph, allowing for bi-directional message passing between specific and broad GO terms. It is important to note that we do not specify the type of reversed edges which is different from prior work (Chen *et al.*, 2022), such as “parent_is_a” or “parent_part_of”. This enables the GO DAG encoder to concentrate exclusively on capturing the broad hierarchical relationships between pairs of nodes, irrespective of the particular hierarchy types involved. This modification contributes to a more effective encoding of hierarchical relationships. The GO specificity knowledge graph created in this manner is shared among all subsets, mimicking the curation scenarios in real-world situations where human curators refer to a part of the GO DAG during GOA consistency checks.

Retrieval of existing GOA

We retrieved existing GOA from the `NCBI-gene2go` resource as a type of background knowledge of each GOA instance in the dataset. Consider the GOA instance indexed by a triplet of identifiers, “GO:0015804_Gene:836729_PMID:27925655”. We retrieved and added a list of GO terms to this instance if they are existing annotations of “Gene:836729” and supported by either the same or other direct literature, such as “GO:0015175”, “GO:0005774”, and “GO:0015173”.

3.3 Modeling

We extended a previously proposed model, which involves the direct fine-tuning of PubMedBERT (Gu *et al.*, 2021; Chen *et al.*, 2022), on the training and development set. Our study extended this model, integrating the sourced background knowledge either as input or within the inconsistency detection model. As a baseline, we adopted a random guess strategy that assumes a normal distribution for all categories of inconsistencies.

Pre-encoding of existing GOA pattern and GO specificity knowledge

We employed the DGL toolkit (Wang *et al.*, 2019) to model GOA as an undirected heterogeneous graph (Fig 2). We represented each instance as a pair of two connected nodes, where one node is a tuple of PMID and Gene (PMG), and the other node is a corresponding GO term. Edges exist only between PMG and GO term nodes, which represent an annotation relation. The local topology of the modeled GOA graph can be used to identify existing GOA patterns. For example, Fig 2 illustrates the co-annotation patterns of “GO:0015175” and “GO:0015804” across “Gene:836729” and “Gene:818801”, supported by “PMID:27925655”. The co-annotation of GO suggests that the two genes are highly related in their function. In fact, they are members of the same gene family that encodes transmembrane amino acid transporter proteins, based on information from Entrez Gene (Brown *et al.*, 2015). The identification of annotation patterns is crucial in detecting GOA inconsistencies. To achieve this, we set up a learning objective to classify the edge type on the GOA heterogeneous graph using a GNN model designed to automatically learn the local topological features in existing GOA.

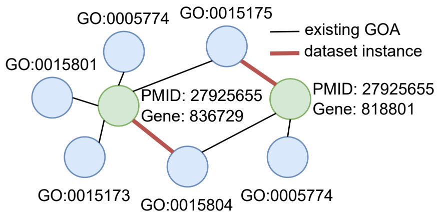

Fig. 2. Modeling two GOA instances as an un-directed heterogeneous graph

We constructed three heterogeneous knowledge graphs based on the GOA inconsistency subsets, including training, development, and held-out test. To label the edges in our study, we employed the following approaches: An edge connects a PMG and a GO term node, representing either a consistent or an inconsistent GOA instance. Consistent GOA instances are labeled as "annotate" edges, whereas inconsistent GOA instances, irrespective of the type of inconsistency, are labeled as "not_annotate" edges. Additionally, existing GO terms that serve as background knowledge for each individual GOA instance are labeled as "annotate" edges with the PMG node. After conducting multiple trials, we determined that the optimal initialization of node representation is 8-dimensional all-ones vectors for PMG nodes and random 8-dimensional vectors for GO term nodes.

We designed a GNN structure with three HeteroGraphConv layers (Wang *et al.*, 2019) to encode existing GOA patterns into node embeddings (Figure 3). A HeteroGraphConv layer comprises two aggregation steps, an activation function, and batch normalization. The initial step consists of two GraphSAGE sub-layers that are responsible for aggregating information propagated through neighboring nodes of the same edge type. The second step involves aggregating the output of the two GraphSAGE sub-layers using a convolution function. This approach is specifically devised for inductively training node features across diverse graphs. We use the rectified linear unit (ReLU) as the activation function for each HeteroGraphConv layer, followed by batch normalization.

To enable our implemented GNN for edge type classification, we added a ~~single-layered~~ multi-layer perception with a single fully-connected hidden layer (MLP) at the end to form classification head. The input to the MLP is two concatenated 8-dimensional node vectors, representing the vectorization of an edge between a pair of nodes. The output of the MLP is a 2-dimensional logit vector. We apply the argmax function to the logit vector, predicting the type of edge as either "annotate" or "not_annotate". Following the training of the knowledge graph constructed from the training and development GOA inconsistency dataset, when in evaluation, we apply the pre-trained GNN to the held-out test set to obtain the node embeddings of PMG and GO terms. We retrieve the node embeddings from the output of the last HeteroGraphConv layer.

We encoded GO specificity knowledge into 16-dimensional node embeddings using a similar approach proposed in previous research (Chen *et al.*, 2022). However, we made a slight alteration by merging the "is_a" and "part_of" edges into one type, "children_of", and the "parent_is_a" and "parent_part_of" edges into "parent_of". This modification was implemented because we observed that the previous GNN model could potentially exploit the type of reversed edges in the pre-training objective. For instance, the reversed "parent_is_a" edge from "behavior" to "feeding behavior" could aid the GNN model in predicting the "is_a" edge type in the opposite direction. This merging of edge strategy is appropriate for training the GNN model to incorporate GO specificity knowledge as it does not alter the hierarchical relationships between paired GO terms.

Integration of background knowledge in the input

- GeneRIF substitution: We performed fine-tuning of a new PubMedBERT model on instances where the evidence texts were substituted with GeneRIF (Gene Reference Into Function) statements.
- Entity reformulation: We performed fine-tuning of a new PubMedBERT on instances where mentions of biological concepts in the input were pre-extracted and reformulated using linguistic markers.

Integration of background knowledge within model

We developed a joint model, referred to as GNN-PubMedBERT (shown in Figure 4), which integrates the pre-trained topological features of ontology and annotation with distributional semantics for automatic GOA inconsistency detection. This integration is achieved by flat concatenation of node embeddings and the last hidden layer of PubMedBERT, which serves as the input for a single-layered MLP to form a multi-class classifier. The design of the joint model allows for different combinations of topological feature integration, such as GO DAG, existing GOA, or a combination of both (referred to as **GoDg & exGOA**).

We extended PubMedBERT model with an implementation of an entity encoding layer (or entity embeddings) to process the input instances (Fig 4). This layer encodes the text spans of pre-extracted biological concepts using consecutive 1s and other input tokens using consecutive 0s. For example, assuming an input consists of three tokens: "ClpXP", "degradation", "machine", the corresponding entity encoding will be $(1, 1, 0)$. As "ClpXP" is a gene, "degradation" is a GO term, while "machine" is not a biological concept. Fig 4 demonstrates a GOA instance with a four-layered encoding, including entity embeddings, token embeddings, sentence embeddings, and positional embeddings. The entity embeddings is stacked with the other three encodings, forming the complete input representation for the model.

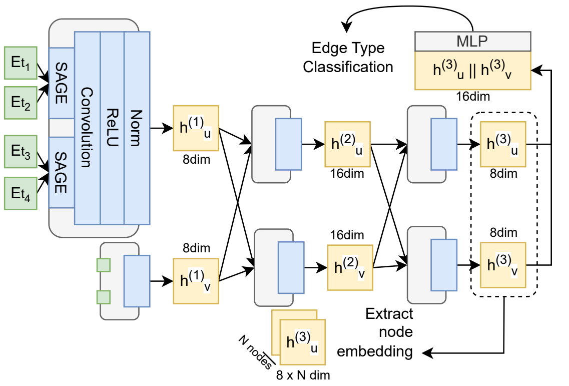

Fig. 3. Architecture of Graph Neural Network (GNN) with the objective of edge type classification for encoding existing GOA patterns. Et_n denotes the initial vectorization of node n representation. h_u^n denotes the hidden representation of node u in the nth layer of GNN; SAGE denotes the GraphSAGE sub-layer for aggregation of node features passed through same type of edges; Convolution ~~aggregate~~ aggregates output of SAGE sub-layers; ReLU denotes the Rectified Linear Unit, which is the activation function; Norm denotes batch normalization; || denotes the flat concatenation of two node vectors; MLP denotes ~~single-layered~~ a multi-layer perceptron with one hidden layer.

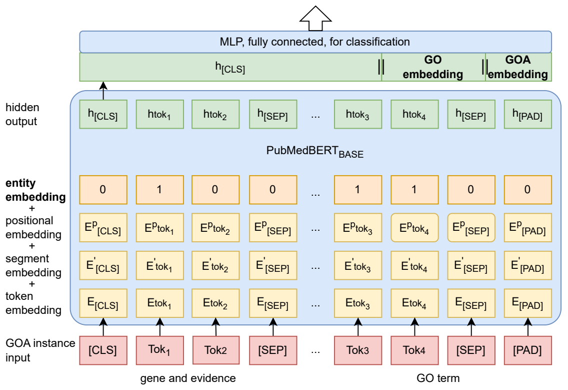

Fig. 4. Architecture of joint model for GOA inconsistency detection, with the addition of entity layer in the input. Tok_* denotes a linguistic token, E_* and h_* denote a token embedding, [CLS] and [SEP] are special tokens marking the boundary of an input pair. The highlighted entity embedding layer illustrates a case where tok_1, tok_3, and tok_4 are part of the pre-extracted biological concepts and thus were encoded in digit 1. This figure demonstrates the combination of $\text{h}_{[cls]}$, GODAG, and GOA KG.

3.4 Metrics

Evaluation follows a one-vs-all strategy for the detection of each specific type of inconsistency, e.g. for the over-specific label, we assume positives are instances with the over-specific label and negatives are instances with any other label. In this case, True positives (TP), False positives (FP), False negatives (FN) are counted as follows:

- TP: The predicted label is over-specific for a positive instance
- FP: The predicted label is over-specific for a negative instance
- FN: The predicted label is not over-specific for a positive instance

We use *Precision* ($P = \frac{TP}{TP+FP}$), *Recall* ($R = \frac{TP}{TP+FN}$), and F_1 score ($F_1 = 2 \times \frac{P \times R}{P+R}$) as evaluation metrics.

3.5 Experiments

We conducted a set of experiments to examine the effectiveness of integrating background knowledge through two approaches: either incorporating it into the input or embedding it within the model. In particular, we compare the performance of different types of background knowledge integration alternatives (as summarized in Table 1). As a baseline, we use a naive strategy of random guessing (**baseline**), which could theoretically achieve an average precision, recall, and F-score of 0.20, given that there are five classes with 500 instances in each for evaluation. The fundamental competitor model is direct fine-tuning of **PubMedBERT** without any integration of background knowledge. We also fine-tuned PubMedBERT on instances where literature evidence was replaced by GeneRIF statements (**+GeneRIF**) to determine whether these statements, which include concise gene function descriptions, were suitable for automatic GOA inconsistency detection. Furthermore, we fine-tuned another PubMedBERT on instances that had pre-extracted biological concepts and process the instances using entity embedding extensions (**+Entity Embedding**). We then examined the individual (**+GoDg / +exGOA**) and combined integration of GODAG and existing GOA (**+GoDg & exGOA**) using the GNN-PubMedBERT model. In addition, we generated a set of dummy node embeddings (**+dummy embeddings**) in a Gaussian distribution, serving as a baseline for the evaluation of graph knowledge integration. This allows us to determine whether the integration of pre-trained graph knowledge embeddings in the GNN-PubMedBERT model was effective or not, compared to the dummy node embeddings.

Approach	Description
baseline	Random guess strategy assuming normal distribution for all types of inconsistencies
PubMedBERT	Direct fine-tuning of PubMedBERT without the integration of any sourced background knowledge as multi-type single-class GOA inconsistency classifier
+ GeneRIF	(PubMedBERT+) Substitution of original literature title and abstract evidence using GeneRIF statements
+ Entity Embedding	Addition of entity encoding layer in PubMedBERT to process GOA instances with pre-extracted biological concepts
+ Entity Reformulation	Addition of linguistic markers to input text to mark the text spans of pre-extracted biological concepts
+ GoDg	Transferring GO specificity knowledge using GNN-PubMedBERT
+ exGOA	Transferring existing annotation patterns using GNN-PubMedBERT
+ GoDg & exGOA	Combining the transferred GO specificity knowledge and existing annotation pattern using GNN-PubMedBERT
+ dummy embeddings	Random generation of embeddings in normal distribution as independent regularizer to PubMedBERT

Table 1. Summary of the experimented approaches

4 Results

Table 2 provides a summary of the experimental outcomes. Overall, each system with variation in leveraged background knowledge demonstrates strong effectiveness over the baseline. This indicates that automatic approaches are apt for detecting inconsistencies in GOA, demonstrating their ability to make informed and reliable predictions that surpass random chance.

The joint model, GNN-PubMedBERT for integrating graph-structured background knowledge, stands out by combining the integration of GO DAG and existing GOA. It notably outperforms other background knowledge integration approaches, such as +GeneRIF, +EntityEmbedding, and +EntityReformulation, in detecting three out of the four most prevalent types of inconsistencies (excluding irrelevant GO mentions). This underscores the effectiveness of the deployed strategy of joint GNN-PubMedBERT in integrating background knowledge, further supporting its success in improving the detection of GOA inconsistencies.

Utilizing GeneRIF statements as an additional source of background knowledge has demonstrated a notable enhancement in *Recall* ($+0.14$) for the detection of IG type inconsistencies comparing with the competitor model (PubMedBERT). However, it yields a slightly lower F_1 (-0.01), particularly in detecting other types of inconsistencies, with a decrease of -0.22 in F_1, especially for over-specific inconsistencies.

Utilizing entity embeddings (+Entity Embedding) for the integration of biological concepts results in slightly better performance compared to in-text reformulation using linguistic markers (+Entity Reformulation), except for the identification of irrelevant GO mentions (IM) ($0.88 < 0.94$ in F_1). This approach does not improve the detection of self-consistency (PubMedBERT F_1 =0.43 cf. +EntityEmbedding F_1 =0.40 or +EntityReformulation F_1 =0.39).

Incorporating the GO DAG (+GoDg) as an additional crucial source of background knowledge improves the detection of self-consistent($+0.20$ in F_1), over-specific($+0.03$ in F_1), and over-broad inconsistencies($+0.05$ in F_1) as compared to PubMedBERT. However, this integration does not improve the identification of inconsistencies related to irrelevant GO mentions (IM) and incorrect genes (IG), aligning with the findings drawn in previous research using GNN-BERT (Chen *et al.*, 2022).

On the other hand, the integration of existing GOA(+exGOA) enhances the detection of all four prevalent types of inconsistencies when compared to both the fundamental competitor (PubMedBERT) and the integration of the GO DAG (+GoDg). Despite this improvement, entity reformulation continues to outperform +exGOA, particularly in the detection of irrelevant Gene Ontology mentions (IM). Notably, combining two sources of background knowledge, GO DAG and existing GOA (+GoDg & exGOA), exhibits the best performance in detecting most types of inconsistencies among all experiemnted approaches.

The final observation suggests that using dummy embeddings performs better than the baseline competitor, PubMedBERT, in detecting inconsistencies related to self-consistency ($+0.21$ in F_1), over-broad categorizations ($+0.05$ in F_1), and incorrect gene instances ($+0.06$ in F_1). This implies that the inclusion of independently and randomly

	consistent (CO)			over-specific (OS)			over-broad (OB)			irrelevant GO mention (IM)			incorrect gene (IG)		
	Precision	*Recall*	F_1	*Precision*	*Recall*	F_1	*Precision*	*Recall*	F_1	*Precision*	*Recall*	F_1	*Precision*	*Recall*	F_1
baseline	0.20	0.20	0.20	0.20	0.20	0.20	0.20	0.20	0.20	0.20	0.20	0.20	0.20	0.20	0.20
PubMedBERT	0.42	0.44	0.43	0.70	0.65	0.68	0.73	0.72	0.73	0.87	0.91	0.89	0.62	0.61	0.62
+ GeneRIF	0.41	0.39	0.40	0.67	0.35	0.46	0.67	0.71	0.69	0.79	0.85	0.82	0.54	0.75	0.63
+ Entity Embedding	0.46	0.35	0.40	0.65	0.73	0.69	0.75	0.72	0.74	0.88	0.88	0.88	0.61	0.71	0.66
+ Entity Reformulation	0.50	0.32	0.39	0.64	0.73	0.68	0.69	0.77	0.73	0.93	**0.94**	**0.94**	0.60	0.67	0.63
GNN-PubMedBERT															
+ GODAG (GoDg)	0.60	0.67	0.63	0.65	0.77	0.71	**0.82**	0.75	0.78	0.87	0.87	0.87	0.71	0.55	0.62
+ exGOA	0.76	0.73	0.75	0.76	0.70	0.73	0.80	0.76	0.78	0.89	0.92	0.90	0.65	0.75	0.70
+ GoDg & exGOA	**0.82**	**0.89**	**0.86**	**0.80**	**0.78**	**0.79**	0.79	**0.81**	**0.80**	**0.97**	0.85	0.91	**0.72**	**0.75**	**0.74**
+ dummy embeddings	0.62	0.67	0.64	**0.80**	0.58	0.68	0.75	0.81	0.78	0.85	0.92	0.88	0.68	0.69	0.68

Table 2. Performance comparison of systems variants considering the integration of different background knowledge alternatives for detecting GOA inconsistencies on a test set. The baseline and PubMedBERT approaches did not utilize any background knowledge, while the combined GoDg & exGOA approach incorporated both GO specificity and existing GOA. Bold font is used to indicate the highest score in each column.

generated dummy embeddings serves as a regularization technique for the PubMedBERT model, leading to improved generalization in detecting inconsistencies. Furthermore, it indicates that the model's effectiveness is still limited by the quality and quantity of the available data.

5 Discussion

In this paper, we investigated the value of utilizing biological background knowledge and developed several approaches to incorporate such knowledge to facilitate the automatic detection of GOA inconsistencies. Here, we discuss both the strengths and limitations of each approach employed in integrating background knowledge during the experiments. Additionally, we discuss the impact of the newly introduced test set on our study and demonstrating its reliability for evaluation purpose as compared to the original BC4GO-derived test set. We present two ad-hoc examples of inconsistencies detected by the model in the real-world gene2go dataset, illustrating its practical utility on real-world GOA instances.

The results of PubMedBERT as compared with +GeneRIF in Table 2 reveal that, GeneRIF statements are a more dependable alternative evidence source for detecting instances of incorrect gene selection (IG) than leveraging title and abstract of direct literature as evidence. This preference stems from the observation that literature abstracts often reference multiple genes, introducing confusion for both human and automated curation systems when identifying the correct gene for annotation. In contrast, GeneRIF typically presents the functional context for a single gene product. This enables automatic inconsistency detector to categorize an instance as IG without the distraction of other gene mentions if the annotated gene product is absent from the GeneRIF statements. This strategy notably improves *Recall* in the detection of IG. However, relying solely on GeneRIFs as evidence risks losing essential information for detecting other types of inconsistencies, especially resulting in a significant drop in *Recall*(-0.43 cf. +GoDg & exGOA) for identifying “over-specific” annotations. This suggests that GeneRIF may not offer adequate contextual cues for our implemented language models to distinguish specificity nuances between GO terms.

However, our model currently faces challenges in distinguishing between related gene products that present as highly similar text strings in the evidence text, exemplified by difficulties (comparing column IG to other columns in Table 2) in differentiating between entities like the two protein complexes “mTORC1” and “mTORC2”. For instance, the detector struggles to identify inconsistency between “mTORC2” and a GeneRIF statement such as “Glutamine synthetase limits beta-catenin-mutated liver cancer growth by maintaining nitrogen homeostasis and suppressing mTORC1”, leading to a decrease in *Precision* in IG detection. We hypothesize that integrating background knowledge to differentiate between gene products with highly similar text strings could offer a viable solution. The integration of existing GOA resulted in a substantial improvement in IG detection, likely attributed to the nuanced understanding of related genes provided by the knowledge graph formed by existing GOA. This integration generates diverse node embeddings that prove instrumental in identifying incorrect genes. Despite this improvement, the accessibility of GeneRIF statements, in contrast to the resource-intensive process of pre-training node embeddings on existing GOA knowledge graphs, underscores the continued value of GeneRIF as an evidence source.

Pre-extracted biological concepts have traditionally been favored for the processing of textual information. Many curation-assisting tools currently rely on keyword highlighting, such as PubTator (Wei *et al.*, 2019). However, it is unclear whether this approach, pre-identification of key concepts as keywords, is necessary for automatic systems as well. Our experiments demonstrate that PubMedBERT, which utilizes pre-trained distributional semantics and self-attention mechanisms, is capable of identifying and aligning biological concepts within raw text without pre-extraction. Compared to entity embedding extension (+Entity Embedding) and entity reformulation, PubMedBERT performs consistently well in detecting most types of prevalent inconsistencies. Nonetheless, pre-extraction of biological concepts may still have some value, as it can help to reduce the variances in gene ontology representations. For example, in cases where “intake of food” and “feeding behavior” are co-mentioned in the same text but the latter is marked as an irrelevant GO term annotation, PubMedBERT may fail to detect the inconsistency as it cannot align these phrases as the same GO concept. However, entity reformulation, which employs special linguistic markers, can partially standardize various textual representations of GO concepts, allowing the model to generalize alignment across different forms of GO term representations. We posit that pre-extraction of biological concepts may not be essential for BERT-based models if other strategies, such as using sufficient training data or pre-normalization of entities, can accommodate the variances in GO term representations.

Our findings substantiate the proposition that a nuanced comprehension of GO specificity, coupled with the utilization of existing GOA, is crucial for identifying inconsistencies. By incorporating the GO DAG, the detection of inconsistencies related to GO specificity has improved significantly, resulting in the achievement of the highest *Precision* in identifying over-broad inconsistency. However, the integration of GO DAG still faces challenges in differentiating subtle semantic differences between highly related GO terms. This is revealed from the detection of over-specific inconsistencies as worse than OB. Specifically, the synthesis of over-specific inconsistency involves capturing more nuanced semantic differences, specifically focusing on direct children, in contrast to the broader scope of over-broad inconsistency, which encompasses all

ancestors. Furthermore, the integration of GO DAG has led to an excessive sensitivity in flagging OS to suspicious instances, as errors arise from misidentifying IM as OS if the irrelevant GO mention is a direct child of the correct GO term annotation.

The incorporation of existing GOA into the detection process improves the identification of all prevalent types of inconsistencies. Surprisingly, we observed that this background knowledge surpasses the GO DAG in detecting over-specific inconsistency, with an increase in *Precision* of 0.11. This is due to the fact that the knowledge of GO specificity is also embedded in existing GOA. Specifically, a more general GO term is likely to be linked to numerous PMG nodes, whereas a more specific term is likely to be linked to a smaller number of PMG nodes. However, the accuracy of this integration can be influenced by the distribution of GO terms used in the annotations. We noted that the model tends to make overly broad predictions for frequently appearing GO terms such as "protein binding" or "process", resulting in a decrease in *Precision*. In contrast, GO DAG is not affected by the annotation distribution. Similarly, we observed that the model with the integration of existing GOA tends to only predict rarely appearing GO terms as OS, while many over-specific GO terms that are moderately distributed in existing annotations remain unidentified. We argue that while integrating with existing GOA outperforms GO DAG in detecting GOA inconsistencies, the reliability of this approach depends on the distribution of annotations within the training set. This background knowledge is not suitable for identifying inconsistencies if extremely broad or specific GO terms are used.

The combination of both GO DAG and existing GOA may potentially alleviate the discussed limitations in either one as the single background knowledge. We found this method can better distinguish semantic nuance, referring to the reduced gap of F_1 between the detection of OS and OB (0.01). This method improves the detection of IM and IG inconsistency while sole integration of GO DAG is not capable. It also lifts the *Recall* in detection of OB without sacrificing the *Precision*, contrasting to the sole integration of existing GOA. Similarly, this approach, regulated by the GO DAG, does not make over-specific predictions of rarely appearing GO terms. It lifts the *Precision* in detection of OS without sacrificing the *Recall*. We assume that certain self-regulation rules are generalized through the combination of multiple types of background knowledge on domain-specific semantic inconsistency detection task.

A key finding in our study is that the use of dummy embeddings outperformed the fundamental competitor (PubMedBERT) in the detection of self-consistency, OB, and IG instances. We attribute this improvement to two reasons: firstly, the dummy embeddings may have acted as an independent regularizer for PubMedBERT, preventing the model from over-fitting during fine-tuning on limited data. Secondly, it may have reduced the impact of noisy samples during fine-tuning, where a noisy sample refers to a consistent instance being incorrectly labeled as an inconsistency or vice versa. Although the dummy embeddings achieved the same F_1 as integration of GO DAG and existing GOA in the detection of OB, it resulted in a low *Precision*. Similar trade-offs between *Precision* and *Recall* were also observed in the detection of IM. We suspect that because the dummy embeddings are not providing any informative support when compared with GO DAG and existing GOA. The achievements made by dummy embeddings will fade away if human curators become involved in expanding the existing dataset with higher-quality instances. We also encourage the exploration of alternative independent regularizers to further improve the model's generalization capability.

Another contribution of this study is the introduction of a novel test set designed for evaluating inconsistency detection in GOA, addressing several limitations associated with existing methodologies. Firstly, our decision to create this new test set comes from the acknowledgment of significant annotation discrepancies among annotators in BC4GO (Van Auken *et al.*, 2014), rendering it an unreliable source for simulating real-world GOA inconsistencies. In addition, only 11.5% of evidence sentences annotated in BC4GO were extracted from the title and abstract of literature sources. The majority of the evidence sentences were extracted from the results and discussion section within full-text article. Acknowledging the real-world challenges associated with accessing full articles, our approach deliberately focus on utilizing evidences that were completely extracted from the title and abstract.

To further improve the reliability of our evaluations, we developed a held-out test set derived from the NCBI `gene2go` database. This test set remains independent of existing training and development subsets, as evidenced by the absence of overlap in literature PMID sources. Realizing the importance of authentic inconsistencies, we adopted the peer-reviewed inconsistency synthetic strategy proposed in (Chen *et al.*, 2022) for test set generation, followed by a manual check.

Our experimental findings demonstrate that the PubMedBERT model outperforms the BC4GO-derived dataset on this new test set, providing a more realistic evaluation of model performance on real-world data. This comprehensive approach aims to address the limitations associated with existing test sets and contributes to a more robust assessment of GOA inconsistency detection models.

To achieve a more comprehensive understanding of the reliability of our results, we directly applied the detector (+GoDg & exGOA variant) on the real-world gene2go dataset. Table 3 displays two detected inconsistencies observed during our experiment using the detector. While the precise reasons for the model flagging these instances as inconsistencies remain unknown due to the current lack of sufficient explainability in our approach, we may speculate on the following potential reasons. The first flagged instance is categorized as "over-specific". This may due to that the evidence supporting the annotation is speculative, and the inconsistency detector concludes that the selected GO term conveys excessive information. The second flagged instance is flagged as "over-broad". We found when changing the associated GO term to the more specific "regulation of multicellular organism growth (GO:0040014)" can be more accurately describe the gene function conveyed in the literature evidence. The proposed synthetic dataset offers a controlled environment for exploring and assessing methods for detecting inconsistencies in GOA. This is a necessary precursor to investing in the development of inconsistency in real-world data. Given the current absence of datasets containing known real-world GOA inconsistencies, and the ongoing challenge of discovering such inconsistencies, our plan is to collaborate with expert curators in the future. Together, we aim to create a comprehensive dataset through integration of real-world inconsistencies and systematically evaluate our approaches.

In summary, our study has made significant contributions by presenting a comprehensive exploration of various biological background knowledge and delivering novel approaches for their integration in automatic GOA inconsistency detection (Outlined in Figure 1). Through our research, we have identified distinct values in each approach or their synergistic combination, emphasizing the role of background knowledge in enhancing semantic inconsistency detection. Specifically, our methodologies effectively leverage the distributional semantics of GOA instances and the topological features of their associated background knowledge.

6 Conclusion

Our study demonstrated the advantage of integrating biological background knowledge for automatic detection of semantic inconsistency in GOA. We investigated various types of background knowledge and proposed multiple approaches to their integration, leveraging distributional semantics in GOA and topological information in the knowledge graph.

Gene: VHA-19
Evidence: Critically, VHA-19 is expressed in the excretory cell in both larvae and adults, suggesting that it **may have a role** in osmoregulation in *C. elegans* more generally, **probably** in trafficking or secretion pathways. (PMID:22768351)
GO Term: intracellular protein transport (GO:0006886)
Inconsistency: over-specific

Gene: NPRL-2
Evidence: Here, we report that a mmBCFA-derived sphingolipid, d17iso-glucosylceramide, is a critical metabolite in **regulating growth and development**. Further analysis indicated that this lipid function is mediated by TORC1 and antagonized by the NPRL-2/3 complex in the intestine. Strikingly, the essential lipid function is bypassed by activating TORC1 or inhibiting NPRL-2/3. (PMID:23705068)
GO Term: regulation of multicellular organismal process (GO:0051239)
Inconsistency: over-broad

Table 3. Examples of two detected inconsistent GOA instances in gene2go

Our models showed significant improvements in detecting specific types of GOA inconsistencies compared to the current state-of-the-art (Chen *et al.*, 2022). We found combining the integration of both GO specificity knowledge and existing annotation patterns using joint GNN-PubMedBERT model showcased the greatest effectiveness in the detection of prevalent inconsistencies.

In the future, we plan to collaborate with expert curators to conduct systematic qualitative evaluations. We will explore ensemble models that unify our proposed approaches into a comprehensive inconsistency detection system. Finally, we will apply an end-to-end GOA inconsistency system to biological databases and assess its implications for biological database curation processes.

References

Aronson, A. R. (2001). Effective mapping of biomedical text to the umls metathesaurus: the metamap program. In *Proceedings of the AMIA Symposium*, page 17. American Medical Informatics Association.

Ashburner, M. *et al.* (2000). Gene ontology: tool for the unification of biology. *Nature Genetics*, **25**(1), 25–29.

Balakrishnan, R. *et al.* (2013). A guide to best practices for gene ontology (GO) manual annotation. *Database*, **2013**.

Bateman, A. *et al.* (2021). UniProt: the universal protein knowledgebase in 2021. *Nucleic Acids Research*, **49**(D1), D480–D489.

Bauer, S. (2017). Gene-category analysis. *Methods in Molecular Biology (Clifton, NJ)*, **1446**, 175–188.

Binns, D. *et al.* (2009). QuickGO: a web-based tool for Gene Ontology searching. *Bioinformatics*, **25**(22), 3045–3046.

Brown, G. R. *et al.* (2015). Gene: a gene-centered information resource at ncbi. *Nucleic Acids Research*, **43**(D1), D36–D42.

Bult, C. J. *et al.* (2019). Mouse genome database (MGD) 2019. *Nucleic Acids Research*, **47**(D1), D801–D806.

Camon, E. B. *et al.* (2005). An evaluation of go annotation retrieval for biocreative and goa. *BMC Bioinformatics*, **6**(1), 1–11.

Carbon, S. *et al.* (2021). The gene ontology resource: enriching a GOld mine. *Nucleic Acids Research*, **49**(D1), D325–D334.

Chen, J. *et al.* (2021). Automatic consistency assurance for literature-based gene ontology annotation. *BMC Bioinformatics*, **22**, 565.

Chen, J. *et al.* (2022). Exploring automatic inconsistency detection for literature-based gene ontology annotation. *Bioinformatics*, **38**(Supplement_1), i273–i281.

Chen, Q. *et al.* (2017a). Benchmarks for measurement of duplicate detection methods in nucleotide databases. *Database*.

Chen, Q. *et al.* (2017b). Duplicates, redundancies and inconsistencies in the primary nucleotide databases: a descriptive study. *Database*, **2017**.

Dimmer, E. C. *et al.* (2010). Formalization of taxon-based constraints to detect inconsistencies in annotation and ontology development. *BMC bioinformatics*, **11**(1), 1–10.

Faria, D. *et al.* (2012). Mining GO annotations for improving annotation consistency. *PLoS One*, **7**(7), e40519.

Funk, C. *et al.* (2014). Large-scale biomedical concept recognition: an evaluation of current automatic annotators and their parameters. *BMC Bioinformatics*, **15**(1), 59.

Gilks, W. R. *et al.* (2002). Modeling the percolation of annotation errors in a database of protein sequences. *Bioinformatics*, **18**(12), 1641–1649.

Gobeill, J. *et al.* (2008). Gene ontology density estimation and discourse analysis for automatic generif extraction. In *BMC bioinformatics*, volume 9, pages 1–10. BioMed Central.

Goudey, B. *et al.* (2022). Propagation, detection and correction of errors using the sequence database network. *Briefings in bioinformatics*, **23**(6), bbac416.

Gu, Y. *et al.* (2021). Domain-specific language model pretraining for biomedical natural language processing. *ACM Transactions on Computing for Healthcare (HEALTH)*, **3**(1), 1–23.

Haendel, M. A. *et al.* (2018). Classification, ontology, and precision medicine. *New England Journal of Medicine*, **379**(15), 1452–1462.

Hamosh, A. *et al.* (2005). Online mendelian inheritance in man (omim), a knowledgebase of human genes and genetic disorders. *Nucleic acids research*, **33**(suppl_1), D514–D517.

Huang, D. W. *et al.* (2009). Bioinformatics enrichment tools: paths toward the comprehensive functional analysis of large gene lists. *Nucleic Acids Research*, **37**(1), 1–13.

Huntley, R. P. *et al.* (2014). Understanding how and why the Gene Ontology and its annotations evolve: the GO within UniProt. *GigaScience*, **3**(1), 2047–217X.

Jonquet, C. *et al.* (2009). The open biomedical annotator. *Summit on translational bioinformatics*, **2009**, 56.

Lu, Z. *et al.* (2006). Finding generifs via gene ontology annotations. In *Biocomputing 2006*, pages 52–63. World Scientific.

Mao, Y. *et al.* (2014). Overview of the gene ontology task at biocreative iv. *Database*, **2014**.

Poux, S. and Gaudet, P. (2017). Best practices in manual annotation with the gene ontology. In *The Gene Ontology Handbook*, pages 41–54. Humana Press, New York, NY.

Škunca, N. *et al.* (2012). Quality of computationally inferred gene ontology annotations. *PLoS Computational Biology*, **8**(5), e1002533.

Tanenblatt, M. *et al.* (2010). The conceptmapper approach to named entity recognition. In *Proceedings of the Seventh International Conference on Language Resources and Evaluation (LREC'10)*.

Thomas, P. D. (2017). The gene ontology and the meaning of biological function. In *The Gene Ontology Handbook*, pages 15–24. Humana Press, New York, NY.

Van Auken, K. *et al.* (2014). BC4GO: a full-text corpus for the BioCreative IV GO task. *Database*, **2014**.

Wang, M. *et al.* (2019). Deep graph library: A graph-centric, highly-performant package for graph neural networks. *arXiv preprint arXiv:1909.01315*.

Wei, C.-H. *et al.* (2019). Pubtator central: automated concept annotation for biomedical full text articles. *Nucleic Acids Research*, **47**(W1),

W587–W593.

Wood, V. *et al.* (2020). Term matrix: a novel gene ontology annotation quality control system based on ontology term co-annotation patterns. *Open Biology*, **10**(9), 200149.
